# The metabolomic profile of a high starch versus no starch diet in athletic horses

**DOI:** 10.1038/s41598-025-23422-z

**Published:** 2025-10-13

**Authors:** Emma Nilsson, Ali A. Moazzami, Jan Erik Lindberg, Anna Jansson

**Affiliations:** 1 Department of Animal Biosciences, Swedish University of Agricultural Sciences, P.O. Box 7011, 750 07 Uppsala, Sweden; 2 Department of Molecular Sciences, Swedish University of Agricultural Sciences, Uppsala, Sweden; 3 Department of Applied Animal Science and Welfare, Swedish University of Agricultural Sciences, P.O. Box 7024, 75007 Uppsala, Sweden

**Keywords:** Diet, Forage, Starch, Metabolomics, Microbiota, NMR, Plasma, Metabolism, Metabolomics, Animal physiology

## Abstract

Feeding a high amount of starch-rich grains is common practice for performance horses even though the horse has evolved to eat a grass based, i.e. low starch diet. To our knowledge, there are no studies using metabolomics to investigate the effects of a high-starch diet in horses. In this study we investigated differences in the plasma metabolic profile of 6 Standardbred horses fed a no-starch, forage-only (F) diet or a high-starch forage-concentrate (FC) diet for 29 days, respectively in a cross-over design. Postprandial plasma samples were collected on the morning of day 25 of each dietary period. Metabolomics analysis of plasma using a targeted ^1^H NMR resulted in the quantification of 52 metabolites. Both a univariate and multivariate analysis of metabolites was performed. The univariate analysis found increased (p < 0.05) plasma concentrations of 2-hydroxybutyrate, citrate, dimethyl sulfone, hippurate, methionine, myo-inositol and proline in diet F and higher concentrations of glycine in diet FC. A PLS-DA analysis could discriminate between diets with good predictive power (Q2 (cum) = 0.745, p = 0.032 in CV-ANOVA). We conclude that diet F was strongest identified by metabolites originating from host-microbial co-metabolism and that the clear metabolomic profile discrimination between diets may have implications for health, performance and behaviour.

**Supplementary Information:**

The online version contains supplementary material available at 10.1038/s41598-025-23422-z.

## Introduction

Feeding a high amount of starch-rich grains is common practice for performance horses^1^ even though the horse has evolved to eat a grass based diet, i.e. a low starch diet, and has a rich microbial flora in the hindgut which effectively ferment plant fibre^2^. The microbes in the hindgut mainly ferment fibre into short-chain fatty acids (SCFA) such as acetate, butyrate and propionate^3^. These SCFA’s are used in the energy metabolism, though there is sparse information about the metabolic fate of these in the horse^4^. Propionate is a gluconeogenic precursor, primarily converted to glucose in the liver and thereafter used as energy^5^. Acetate can be used directly as an energy source by the skeletal muscle during exercise through beta-oxidation^6^ and is used in fat synthesis in the adipose tissue. Butyrate is an energy source for the epithelial cells in the large intestines and may affect the immune system^7^.

Our group has earlier shown that diet, and especially the inclusion of starch-rich cereals instead of forage, may dramatically affect the metabolic and physiological response in athletic horses, both at rest and during exercise^2,8–10^. Energy metabolism is altered with significant changes in, e.g., plasma acetate and insulin levels as well as exercise lactate responses, but also plasma cortisol levels and heart rate seem to be affected^11^. Other studies also indicate that high starch diets are associated with, e.g., altered and stereotypic behaviour^12–14^, rhabdomyolysis^15,16^ and gastric ulcers^17^. The mechanism behind these effects is, however, not clear.

In the studies mentioned above investigating metabolic profiles, only targeted analyses have been used (e.g., immunoassays) and to deepen the understanding of the mechanisms other methods are needed. As far as we know, there are no studies using metabolomics that describe the effects of typical^1^ high-starch diets in athletic horses. Proton nuclear magnetic resonance (^1^H-NMR) and coupled mass spectrometry (MS) have been used to explore the effect of high-cereal inclusion in diets of cows and it was shown to affect the rumen microbiota and increase the concentration of potentially harmful and inflammatory metabolites in rumen^18,19^ as well as metabolites linked to metabolic diseases^20^. In general, the use of metabolomics in horse studies has been sparse compared to other livestock animals such as cows^21^. There are three metabolomics studies on exercising horses where the diet is controlled^22–24^ and two studies investigating dietary effects in sedentary horses, one comparing the urinary metabolome of hay or haylage fed ponies^25^ and another comparing serum metabolome of silage or hay fed horses^26^. Therefore, this study aimed to investigate the effects of a high starch diet on the metabolic profile of athletic horses in a sport horse context, i.e. after road transport to an exercise event. More specifically, we compared the metabolic profiles of plasma from athletic horses fed a no-starch, forage-only diet to those of horses on a high-starch diet, using a cross-over design, targeted metabolomics and a hypothesis generating approach. We hypothesised that horses fed a forage only diet would exhibit a plasma metabolomic profile distinct from horses fed a high starch diet. We expected these differences to be driven by fibre fermentation in the forage only diet and starch digestion in the high starch diet, and we hypothesised to identify both expected differences, e.g. elevated acetate levels in the forage only diet, as well as novel metabolites that could expand our understanding of dietary impacts on equine digestion and metabolism.

## Methods

The experiment was conducted in October to December in 2007 at a clinic and a training camp for harness racing 20 km south of Uppsala, Sweden. The experiment was approved by the Uppsala local ethics committee (C 109/7) and was performed according to relevant guidelines and regulations. The experiment is reported in accordance with the Animal Research: Reporting of In Vivo Experiments (ARRIVE) guidelines.

### Animals, training and housing

Six Standardbred geldings, aged 6.5 ± 0.4 years (mean ± s.d.), with an initial body weight of 515 ± 21 kg (453–584 kg) were used in the study. All horses were in race training and the experimental training protocol included interval (4 × 600 m) and heat training (1,600 or 2,000 m long), which was exactly the same in both periods^10^. The horses were trained two days a week with the aim to keep the horses’ fitness constant throughout the experiment (i.e., intensity and duration were not changed compared to before the experiment). They were housed in individual stalls with wood shavings at night and on days without training, they were kept together in a sand/clay paddock between 08:00 and 15:00 h.

### Experimental design and diets

A cross-over design with two groups of horses (three in each) and two diets fed during two experimental periods of 29 days each was utilized (without any washout period in between). Horses were adapted to the diets for 25 days before sample collection was performed (more details see below). We have earlier shown that significant alterations of the equine colon ecosystem occur within 3 weeks in response to dietary changes^27,28^. Two iso-caloric and iso-nitrogenous diets (that met individual energy and nutrient requirements for very heavy exercise^29^ based on initial body weight) was calculated for each horse. Body condition score was similar on both diets for four of the horses and slightly higher for two horses on diet FC, and mean body weight was 3 kg higher on diet F than on diet FC (earlier published in Jansson and Lindberg^10^). The diets were as follows: 1) a forage-only diet (F) consisting of early-cut grass haylage (timothy, meadow fescue mixture) and 2) a forage-concentrate diet (FC) consisting of late-cut grass haylage (timothy, meadow fescue mixture) supplemented with concentrate containing 35.8% starch (Table 1). The concentrate consisted of 82% oats, 14% soybean meal, 2.7% wheat bran and 1.4% sugar and was given on a 50:50 proportion (forage: concentrate) on dry matter basis (6.3–8.5 kg/day) in diet FC (divided in three meals per day). The forage allowance was 13–17.4 kg/day on diet F and 6.3–8.4 kg/day on diet FC, respectively, which were offered in the afternoon. However, horses had forage leftovers on both diets and in addition performed selective feeding behaviour in diet F (earlier described in Jansson and Lindberg^10^), resulting in a forage: concentrate ratio of 40:60 and 30% higher CP intake on diet FC (Table 2). The chemical composition of the diets is shown in Table 1, feed and leftovers analyses was made using standard methods, earlier described in Jansson and Lindberg^10^, and estimation of metabolizable energy content was made according to Lindgren^30^. The total daily starch intake was 0 and 2503 ± 108 g/day in diet F and FC, respectively. Both diets were supplemented with 51 ± 2 g/day of a vitamin and mineral mix (Miner Röd, KRAFFT, Sweden) and NaCl (36 ± 1 g/day), which was fed together with sugar (180–240 g/day) in diet F to facilitate intake. Due to the calcium: phosphor ratio being below recommendations^29^ in diet FC this diet was also supplemented with 34 ± 1 g/day ground chalk. The supplements were offered together with the concentrate three times a day on diet FC and was divided into two meals for diet F. Diet F was introduced directly on day 1 in both periods. Diet FC was gradually introduced, i.e. on day 1 and 2 the horses received a mixture consisting of 50% F diet and 50% FC diet, thereafter the proportion of diet FC was progressively increased with 10% until reaching full FC ration by day 7. Supplementary Table S1 and S2 presents example diet compositions for both diet F and FC and the main mineral concentrations of the forages.

**Table 1:** Dry matter (%), estimated energy (MJ ME/kg DM) and chemical composition (g/kg DM) of forages and concentrates used in the two diets, a no starch forage-only diet (F) and high starch forage-concentrate diet (FC).

	**Diet F**	**Diet FC**		
	Forage	Forage	Concentrate	Complete FC diet
Dry matter	80	78	90	92
Metabolizable energy	10.4*	8.8	11.4	10,1
Ash	75	56	37	47
Crude protein	104	61	174	118
Neutral detergent fibre	605	600	203	402
Acid detergent fibre	363	370	117	244
Lignin	51	69	27	48
Crude fat	19	15	60	38
Water soluble carbohydrates	79	147		74
Free glucose	35	22	3	13
Free fructose	31	57	0	29
Fructans	4	51	5	28
Starch	0	0	358	179

The early-cut haylage forage for diet F and the late-cut haylage forage, concentrate mixture and complete diet (haylage + concentrate 50:50 DM basis) for diet FC is presented. Table modified from Jansson & Lindberg, 2012^10^.

*Not including the ME from the sugar offered with the vitamin and mineral mix (17 MJ ME/kg DM).

**Table 2:** Daily feed intake (kg) and nutrient (g) and estimated metabolizable energy intake (MJ ME) on the no starch forage-only (F) and high starch forage-concentrate (FC) diet during the 29 days experimental period.

	Diet F	Diet FC
Forage intake	10.07 ± 0.09	4.88 ± 0.09
Concentrate intake	0.29 ± 0.04^a^	7.07 ± 0.04
CP intake^a^	1132 ± 87	1467 ± 66*
NDF intake	6588 ± 507	3885 ± 270*
Starch intake	0	2503 ± 108*
WSC intake	861 ± 66	605 ± 60*
Energy intake^2^	110 ± 6	116 ± 6*

WSC = water-soluble carbohydrates.

Presented as LSmeans ± s.e. Table is modified from Jansson and Lindberg, 2012^10^.

^a^Corresponds to the sugar offered to facilitate intake of the vitamin and mineral mixture.

*Significant difference (p < 0.05) between diet FC and diet F.

Sample collection was performed on day 25, allowing for an adaptation period on both diets. Forage leftovers were removed at 06:00 h, and the horses were kept together in the paddock from 07:30 to 10:30 h. Thereafter, they were returned to their boxes and offered 1 kg of forage (diet F, 8.3 MJ metabolizable energy (ME)) and 1 kg of oats (diet FC, 9.7 MJ ME^31^). We have earlier demonstrated the impact of road transport on the metabolic plasma profile and have suggested that transport should be included in the experimental design if results shall be relevant for competition horses^9^. After the meal, horses were therefore transported 25 km in a trailer to a clinic where a blood sample (10 ml Li-heparinised tubes) was collected (FC: 7.8 ± 2.5 h and F: 7.9 ± 2.6 postprandially). Samples were collected from a catheter in the jugular vein (introduced under local anaesthesia (Carbocain 20 mg/ml, AstraZeneca AB, Sweden) inserted before transport). In one horse, the sample from the forage-only period is missing. The blood samples were kept chilled until centrifugation and the plasma was frozen at -20 ℃ for later analysis.

### ^1^H NMR analysis and sample preparation

A targeted high-throughput ^1^H NMR analysis was performed on the plasma samples, year 2015. Fifty-two metabolites were quantified using NMR spectral data as described below.

Sixty microliters of plasma were filtered using a Nanosep device (3 kDa cut-off, Pall Life Science, Port Washington, NY) to remove proteins, as previously described^32^. The filtrate (40 µL) from the centrifuged samples was combined with phosphate buffer (50 μL, 0.4 mol/L, pH 7.0), water (55 μL), D2O (15 μL), and trimethylsilyl-d4-propionic acid solution (TSP, 10 μL, 5.8 mmol/L). TSP served as an internal standard for metabolite quantification and for monitoring chemical shifts in the NMR spectra. The mixed sample solution (170 μL) was then transferred to a 3 mm NMR tube and processed using a Bruker Avance III spectrometer operating at 600 MHz proton frequency and equipped with a cryogenically cooled probe and an autosampler. A zgesgp pulse sequence (Bruker Biospin) was used to acquire the^1^H-NMR spectrums (25 °C, 512 transients, 4 s relaxation delay, 65 536 data points, 17 942 Hz spectral width;^32^).

The NMR Suite Professional Software package (version 7.5; ChenomX Inc., Edmonton, Canada) was employed to manually correct the spectral baseline and phase. In all spectra, the line-broadening factor was adjusted to ensure that the full width at half maximum of the TSP internal standard signal is 1.0 Hz.

Fifty-two metabolites were identified in the NMR spectra of plasma samples, as previously detailed^32^. Each metabolite was quantified using its specific NMR signal relative to the TSP internal standard. Concentrations were calculated after accounting for interfering signals from other metabolites through an in-house algorithm (Automated Quantification Algorithm, AQuA) implemented in MATLAB (version R2012b, Math Works Inc.), as previously described^32^.

### Statistical analysis

We employed a univariate ANOVA procedure to identify individual metabolites that significantly differed between the dietary treatments. To capture the overall metabolic response and explore patterns that may not be apparent in the univariate procedure, we also performed a multivariate analysis using PLS-DA, which allows for consideration of the correlated structure between metabolites and was well-suited for our hypothesis-generating approach.

All univariate statistical analyses were performed using R (v4.3.1, R Core Team, 2022). Before statistical analysis, values in the metabolomic dataset below the ^1^H-NMR detection limit were imputed with one-fifth of the smallest value detected, and the data were normalized using log transformation. The package DHARMa was used to inspect assumptions for normality. The package lme4 was used to fit a linear mixed-effects model with diet (F and FC) and period (first and second) as fixed effects and horse as a random effect. A model with the interaction between diet and period was tested but no significance was found, and the interaction was therefore excluded to simplify the model while period was kept as a fixed effect to reflect the experimental design. Raw p-values was obtained from an ANOVA procedure and were adjusted for multiple comparisons using the false discovery rate (FDR) method. The significance level for adjusted p-values was set to < 0.05. Nutrient intake was compared using the same model and approach as for the metabolomic data but due to a small number of tests, raw ANOVA p-values below 0.05 was considered significant.

A partial least squares discriminant analysis (PLS-DA) was performed in SIMCA (v17.0.2.34594, Sartorius, 2021) to reveal patterns and see which metabolites drive the separation of diets. All variables were centred and scaled using unit-variance (UV) scaling. A difference or discrimination between periods was not observed in the PCA plot score (data not shown). The variable influence on projection (VIP) scores from the PLS-DA model measures which metabolites are the most important to discriminate between the two diets based on all the components accepted in the model. Metabolites were identified as discriminative if VIP ≥ 1 and VIP jackknife-based confidence intervals (95% CI) were not close to or included zero.

## Results

The ^1^H NMR analysis identified 52 plasma metabolites from horses fed either the no starch forage-only diet or high starch forage-concentrate diet.

### Univariate analysis

No significant effect of period was found (p > 0.05). The ANOVA procedure found 8 plasma metabolites that differed significantly (p < 0.05) after FDR correction between diets (Table 3). Three of the significant metabolites were involved in amino acid metabolism, i.e., the concentration of 2-hydroxybutyrate (p = 0.013), methionine (p = 0.040) and proline (p = 0.013) was higher in diet F compared to diet FC, and glycine (p = 0.013) concentrations were higher in diet FC compared to diet F (Table 3). The metabolite myo-inositol (p = 0.042), which is connected to inositol metabolism, was found in higher plasma concentrations in diet F compared to diet FC, as well as citrate (p = 0.013), which is an intermediate in the TCA cycle (Table 3). In addition, two metabolites linked to host-microbial co-metabolism were also higher in plasma in diet F compared to diet FC (hippurate (p = 0.000) 624% higher and dimethyl sulfone (p = 0.001) 888% higher, Table 3).

**Table 3:** Metabolites that were significant different between diets, no starch forage-only (F) and high starch forage-concentrate (FC).

Metabolite	Related metabolic pathway	Diet		
		F (n = 5)	FC (n = 6)	*p-value*
2-hydroxybutyrate	Amino acid catabolism	5.7 (1.0)	3.1 (0.6)	0.013
Citrate	Energy metabolism	150.5 (31.3)	88.5 (10.9)	0.013
Dimethyl sulfone	Host-microbial co-metabolism	164.0 (50.4)	16.6 (3.7)	0.001
Glycine	Amino acid metabolism	188.6 (31.1)	351.6 (49.9)	0.013
Hippurate	Host-microbial co-metabolism	44.9 (16.5)	6.2 (1.2)	0.000
Methionine	Amino acid metabolism	12.4 (2.4)	7.4 (1.2)	0.040
Myo-inositol	Inositol metabolism	10.7 (2.8)	6.7 (2.2)	0.042
Proline	Amino acid metabolism	76.6 (16.0)	50.3 (10.5)	0.013

Means and standard deviation (SD) are presented in µMol/L and the p-values are after FDR correction.

### PLS-DA analysis and VIP scores

**Fig. 1:**
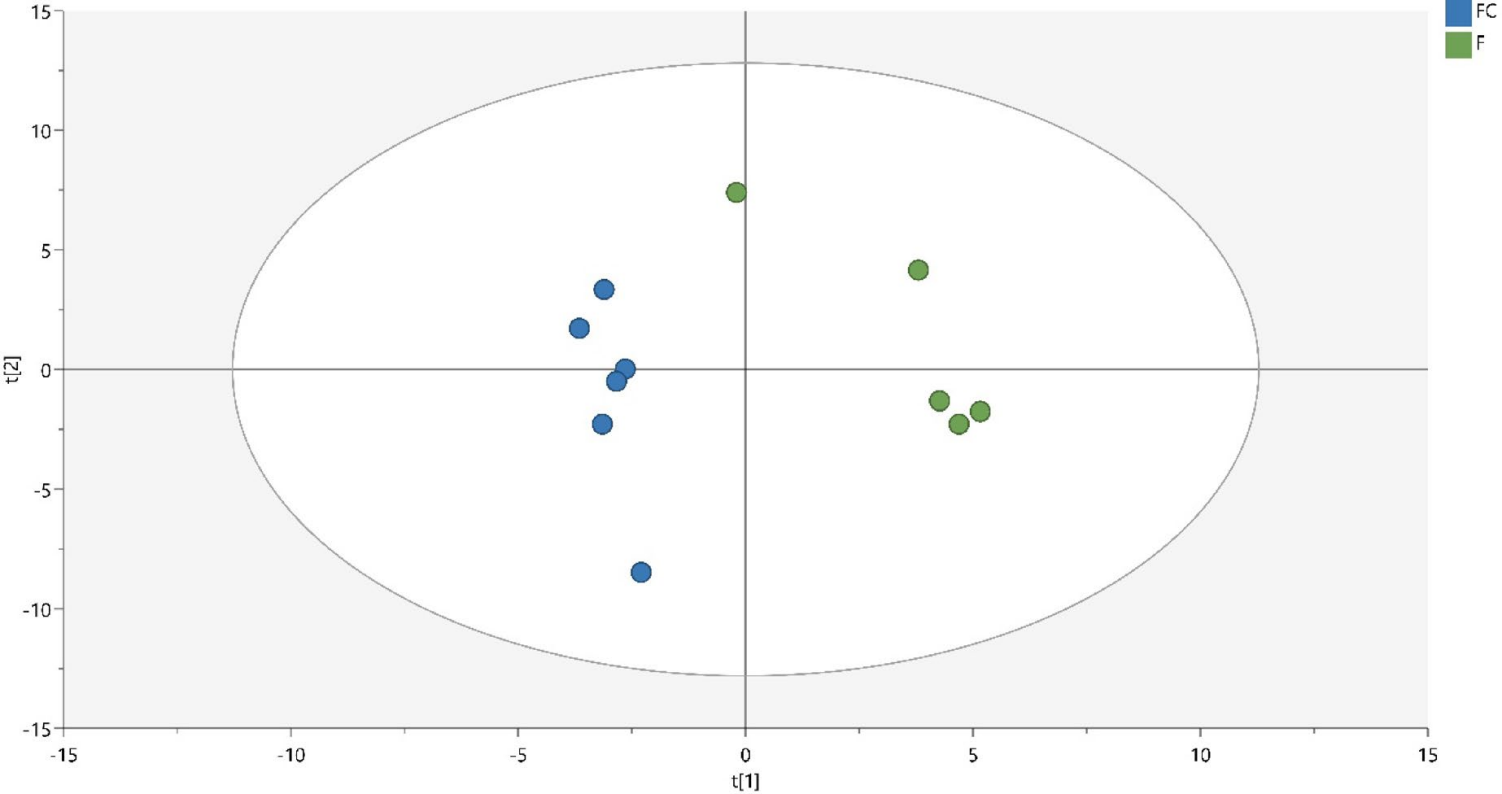
Score plot of the PLS-DA model for plasma metabolites from horses fed a high starch forage-concentrate (FC, blue circles, n = 6) or no starch forage-only (F, green circles, n = 5) diet. Samples from the two diets cluster together and shows a separation along the first predictive component. No outliers were found (all samples within the Hotelling’s ellipse).

The PLS-DA analysis could discriminate between diets with good reproducibility (R2Y (cum) = 0.934), predictive power (Q2 (cum) = 0.745) and CV-ANOVA p-value = 0.032 when building a model with two components and samples clustered together depending on diet (Fig. 1).

The VIP scores for the model identified 18 metabolites of interest (VIP > 1). All 8 metabolites found significant in the univariate analysis were also found discriminative according to their VIP score. Additionally, 2-hydroxyisovalerate, 3 -hydroxybutyrate, acetate, alanine, asparagine, aspartate, carnitine, creatine, propionate and threonine were of importance for the discrimination of diets (Table 4) with plasma concentrations of 2-hydroxyisovalerate, 3-hydroxybutyrate, alanine, propionate, acetate, threonine being higher in horses fed diet F and plasma concentrations of asparagine, carnitine, creatine and aspartate being higher in horses fed diet FC (Table 4).

**Table 4:** Metabolites with a VIP score > 1 calculated from the first component in the PLS-DA model and confidence interval (CI). The higher the score, the more important the metabolite is for separating the no starch forage-only (F) and high starch forage-concentrate (FC) diet.

Metabolite	Related metabolic pathway	Mean (SD)		VIP (CI)
		F (*n* = 5)	FC (*n* = 6)	
Dimethyl sulfone	Host-microbial co-metabolism	164.0 (50.4)	16.6 (3.7)	1.91 (0.47)
Glycine	Amino acid metabolism	188.6 (31.1)	351.6 (49.9)	1.87 (0.83)
Hippurate	Host-microbial co-metabolism	44.9 (16.5)	6.2 (1.2)	1.83 (0.69)
2-Hydroxybutyrate	Amino acid catabolism	5.7 (1.0)	3.1 (0.6)	1.82 (0.69)
Methionine	Amino acid metabolism	12.4 (2.4)	7.4 (1.2)	1.73 (0.97)
Citrate	Energy metabolism	150.5 (31.3)	88.5 (10.9)	1.73 (0.54)
Proline	Amino acid metabolism	76.6 (16.0)	50.3 (10.5)	1.52 (0.99)
Asparagine	Amino acid metabolism	6.1 (1.0)	8.2 (1.4)	1.43 (1.17)
myo-Inositol	Inositol metabolism	10.7 (2.8)	6.7 (2.2)	1.37 (1.12)
3-Hydroxybutyrate	Lipid metabolism	110.4 (38.4)	63.8 (18.2)	1.37 (0.97)
2-Hydroxyisovalerate	Amino acid metabolism	7.1 (2.5)	4.5 (1.2)	1.25 (0.79)
Alanine	Amino acid metabolism	133.4 (20.1)	95.3 (32.5)	1.25 (0.88)
Carnitine	Lipid metabolism	17.5 (2.0)	22.8 (5.1)	1.22 (1.08)
Propionate	Host-microbial co-metabolism	37.8 (13.9)	25.2 (6.3)	1.15 (0.63)
Acetate	Host-microbial co-metabolism	1011.2 (303.1)	744.2 (118.4)	1.15 (0.61)
Threonine	Amino acid metabolism	66.9 (15.6)	53.3 (9.6)	1.06 (0.74)
Creatine	Amino acid metabolism	24.6 (7.2)	37.1 (14.2)	1.05 (0.77)
Aspartate	Amino acid metabolism	17.8 (1.6)	22.2 (5.7)	1.01 (0.56)

Means and standard deviation (SD) are presented in µMol/L.

### Feed intake

Due to the leftovers on both diets the nutrient intake between diet F and FC differed (Table 2). The daily intake of crude protein was higher (p = 0.001) on diet FC than on diet F as well as the calculated intake of metabolizable energy (p = 0.046). NDF intake was higher (p = 0.001) on diet F compared to diet FC. Also, WSC intake was higher (p = 0.001) on diet F. As expected, starch intake was higher (p < 0.001) on diet FC with zero intake on diet F.

## Discussion

Feeding an early cut haylage as in diet F results in horses obtaining their main energy intake from fibre fermentation (as shown by the higher NDF intake on diet F) which produces SCFA, without any starch digestion^3^. In contrast, a cereal based diet like FC, provides a major proportion of the energy through starch digestion in the small intestine, yielding glucose, and from microbial fermentation of starch entering the hindgut, which leads to rapid production of SCFA and lactate^33^. While this knowledge is well established this study is, to the best of our knowledge, the first one using metabolomics technology to investigate the effects of a high-starch diet compared to a forage only diet on the plasma profile of athletic horses, and thus providing a much wider perspective of the dietary effects. The plasma concentration of 8 out of 52 metabolites differed between diets in the univariate analysis, and 18 metabolites were important for diet separation in the PLS-DA analysis. In the following discussion, the focus will be on the metabolites found significant or of importance in either the univariate or the PLS-DA analysis.

The most prominent effect observed in the univariate analysis was the dramatic increase in plasma hippurate and dimethyl sulfone concentrations with diet F. In addition, in the PLS-DA model, diet F was characterized by increased plasma levels of dimethyl sulfone and hippurate as well as 2-hydroxybutyrate, methionine and citrate, while increased levels of glycine, asparagine, carnitine, creatine and aspartate characterized diet FC (Table 4). Hippurate is produced from host-microbial co-metabolism, where gut microbes produce benzoic acid from e.g. polyphenolic compounds, which is taken up into the bloodstream and conjugated with glycine in the liver and kidney to form hippurate^34^. The outer layer of plants is rich in polyphenols^35^ and timothy grass has been found to have high concentration of benzoic acid precursors^36^. We were unable to find information about the phenolic profile of meadow fescue, but it is reasonable to assume that diet F was rich in these compounds and this could possibly explain the high plasma hippurate concentration in horses fed diet F. High levels of hippurate have been associated with a diverse gut microbiome and metabolic health in humans^37,38^. Previous results on the same horses as in the present study showed a more stable faecal microbiota over time on diet F compared to diet FC, horses on the diet F also had lower counts and relative abundance of bacteria associated with laminitis^2^. In the study by Escalona, et al.^39^, which aimed to characterize the metabolome of Standardbred horse’s urine, faeces and plasma, hippurate was detected in urine but not in plasma, and unfortunately, no information on the diet was provided, limiting the determination of dietary influence. However, Leng, et al.^25^ analysed the metabolic profile of urine from hay and haylage fed horses and identified hippurate in both diets. In our study, we did not collect urine samples and can therefore not determine how the diets affected the urinary metabolome, however, we do know that the microbial composition in faeces was altered depending on diet^2^. Considering this, and the big difference seen between diet F and FC, hippurate concentration in plasma may be a possible biomarker of forge intake and could provide insight into the diets of horses in studies, as well as clinical situations, even if no dietary information is available. In fact, hippurate has been proposed as a biomarker in goats’ milk for grazing^40^. However, further research evaluating the relationship between plasma hippurate and forage in horse is needed to investigate its possible use as a biomarker.

Dimethyl sulfone (DMSO_2_), also called methylsulfonylmethane, was also found in high plasma concentration in diet F. It can originate from the degradation of methionine in the gut by microbiota, which produces methanethiol that is taken up in the blood and further metabolised to DMSO_2_, or from dietary origin^41^. Indeed, the plasma methionine concentration was also higher in diet F. There are multiple studies where the physiological effect of DMSO_2_ has been investigated and it is known to have anti-inflammatory and antioxidant properties^42^ and is widely sold as a supplement to humans and equines. Interestingly, an inhibiting effect on cortisol-induced stress in cultured horse muscle cells has been reported when treated with DMSO_2_^43,44^. To our knowledge, DMSO_2_ in blood has previously only been reported in one metabolomic study on horses^22^, and the concentrations (11–15 µmol/L) Bazzano, et al.^22^ observed in a diet of hay and concentrate was very similar to our observation in diet FC (~16 µmol/L) but far below the concentration found in diet F (~164 µmol/L). Moreover, Leng, et al.^25^ found urinary excreted DMSO_2_ in horses fed hay or haylage. In a study on sows, higher levels of plasma DMSO_2_ was observed when feeding a high fibre diet (sugar beet pulp or pectin) compared to a high starch diet^45^. This suggests that a high-forage diet promotes the production of DMSO_2_ which could be of particular interest for high-performing athletic horses, however, the origin of this metabolite in horse plasma needs further investigation.

Besides hippurate and DMSO_2_, additional metabolites differing between diets reflect altered gut microbial activity. The ketone body 3-hydroxybutyrate (BHB) was found in higher plasma concentration in horses fed diet F compared to diet FC. Butyrate (produced by gut microbiota) can be metabolised by the intestines’ epithelial cells, where it is converted to BHB, and if not, it is usually directly metabolised by the liver^7^. BHB is also produced by ketogenesis and can be used as an energy source by tissues and the brain since it can pass through the blood–brain barrier when glucose availability is low^46^. Since horses on the F diet will get more of their energy from SCFA produced in the gut, it is reasonable that they would have more ketone bodies from the beta-oxidation of these lipids in the body. No significant difference was seen between diets comparing acetoacetate and acetone, the other two main ketone bodies. This could support the idea of BHB originating from butyrate metabolism. Another support for this is that horses on diet F had higher counts of *Clostridiaceae* cluster XIVa in faeces, which produces butyrate^2^. An increase of BHB when feeding forage compared to concentrate has also been seen by others evaluating dietary effects on horse metabolism^47^. An interesting effect of BHB in relation to performance is the reported increase in the efficiency of working hearts of rats when infused with BHB (compared to glucose alone, Sato, et al.^48^). If the same effect also exists in horses, this could have positive effects on the performance of horses fed a high forage diet. Interestingly, we have support for improved performance with diet F since the lactate threshold tended to be higher in diet F compared to diet FC (see^10^).

The SCFAs acetate and propionate increased in plasma with diet F. It has been known for long that higher concentrations of grain (starch) in the diet lowers the acetate proportion of SCFA in cecal fluids^3,49^ and that an increase of acetate is dependent on microbial degradation of dietary fibre in the gut. The influence of diet has been well studied in ruminants which show that when a high fibre diet is fed, fibrolytic microbes in rumen will be abundant which mainly produce acetate but if a starch rich diet is fed, the amylolytic microbes will increase which will shift the SCFA production towards propionate and lactate^19,50^. The same pattern has been found in horses when fed a high barley diet, where most changes seems to occur in the colon^51^. In the present study, acetate (and propionate) was important for diet separation in the multivariate analysis, which is in accordance with earlier observations in these horses^10^, i.e., during 24 h, plasma acetate levels were constantly elevated on diet F compared to diet FC, with the exception for during exercise. During exercise, acetate is an energy substrate for skeletal muscle^52^. Overall, the changes in the host-microbial co-metabolism reflect the shift to more fibre being digested in the hindgut on diet F, which is in line with the changes of bacteria found by Willing, et al.^2^. In that study, investigating faecal samples from the same experiment, the F diet decreased the presence of Streptococcus bovis/equinus complex and lactic acid bacteria.

Plasma citrate concentrations were higher in horses fed diet F compared to diet FC. This elevation of citrate and the higher concentration of BHB on diet F are probably due to an increased metabolism of fatty acids, as beta-oxidation produces BHB but also acetyl-CoA, which can enter the TCA cycle and form citrate. Another possibility is that the higher fibre fermentation in the gut on diet F resulted in higher citrate uptake from the forage consumed since plants contain organic acids such as potassium citrate^53^.

There were differences between diets in the plasma concentration of several amino acids. Alterations in the plasma concentration of amino acids are not easy to interpret, as they may depend not only on the dietary intake but also on consumption pattern, digestion capacity and the uptake and release from tissues such as the kidney, liver, red blood cells and muscle. Although diets were calculated to be iso-nitrogenous, true daily crude protein intake was 30% higher in diet FC as horses had forage leftovers (earlier described by Jansson and Lindberg^10^). We can therefore assume that the intake of most amino acids was higher in diet FC than in diet F. However, in a study on horses comparing two forage-only diets with different crude protein contents, no effect was observed on the plasma amino acid profile^54^. In contrast, a study comparing two iso-nitrogenous diets, i.e., a forage-only *vs* forage-cereals-soybean meal diet, the latter showed greater plasma concentrations of methionine, lysine, isoleucine, arginine, glycine and ornithine, and lower concentrations of threonine and alanine^55^. This contrasts with our observations regarding methionine, which was higher on diet F, but in accordance with alanine and threonine responses. One possible explanation for this difference might be a lower soybean meal inclusion in our FC diet compared to the diet used in Graham-Thiers and Bowen^55^, as soybean meal contain more sulphur than oats and methionine is a sulphur containing amino acid. Nevertheless, the interpretation of changes in plasma amino acids needs to be considered in relation to this difference between diets.

In our study, glycine was also a strong driver for the separation of the diets with higher plasma concentrations in diet FC. Glycine is important for synthesising several metabolites such as glutathione, creatine, heme and collagen^56,57^. When increasing the grain inclusion to cows, the glycine concentration in rumen fluid seems to increase^19^. In the study by Bazzano, et al.^22^ where horses were fed a hay and concentrate diet the plasma glycine concentration ( ~125 µmol/L) was in the same range as seen in our study in diet F ( ~189 µmol/L), but far below the concentration seen in diet FC ( ~352 µmol/L). No effect on glycine was found when comparing an early-cut high crude protein forage diet to a late-cut recommended crude protein forage diet^54^, indicating that glycine in our study comes from the grain inclusion. However, the exact origin and implication of this elevated level is still unclear.

Since glycine is one precursor for creatine, the increase in plasma glycine could explain the increase seen in creatine on diet FC. This increase is interesting since creatine supplementation enhances exercise performance in humans^58^. However, ergogenic effects have not been documented in horses^59–61^, possibly due to creatine not being absorbed in the gut or into the muscles efficiently.

As earlier mentioned, plasma methionine concentrations were higher in horses on the F diet compared to the FC diet. Methionine metabolism also produces homocysteine that can be converted to cysteine,^62^. When cysteine is formed, 2-ketobutyrate is produced, which is subsequently degraded to propionyl-CoA and then converted to succinyl-CoA, an intermediate in the TCA cycle. Most methionine will be metabolised to this end^62^. 2-ketobutyrate is reduced to 2-hydroxybutyrate (α-HB), possibly when there is a high NADH/NAD + ratio as during high lipid oxidation^63^. Indeed, we found a higher concentration of α-HB in the plasma of horses fed diet F. α-HB has been investigated as a biomarker for insulin resistance and an indicator of oxidative stress in humans^63^. However, it has also been shown to have an ergogenic effect in mice by increasing the oxidative skeletal muscles’ resistance to fatigue and exercise performance^64^. The relevance of these findings for horses remains to be investigated.

Proline plasma concentrations were also higher in horses fed diet F compared to diet FC. This non-essential amino acid can be synthesized from glutamate, glutamine and arginine and has a unique cyclic structure, making it an important protein backbone, especially for collagen production^62^. In cows, proline concentration increases in rumen fluid with increasing grain inclusion, which stands in somewhat contrast to our result^19^.

The metabolite 2-hydroxyisovalerate, also called 2-hydroxy-3-methylbutyric acid, was found in higher plasma concentration in horses on the F diet compared to the FC diet. This metabolite is produced when valine is transaminated to α-ketoisovaleric acid, which can be further metabolized into succinyl-CoA that can contribute to citrate biosynthesis^62^ and plasma citrate concentrations was indeed higher in horses fed diet F. The elevation of 2-hydroxyisovalerate might indicate catabolic activity (although no difference in valine concentrations was observed between diets).

Methionine, proline, α-HB and 2-hydroxyisovalerate can be transformed into succinyl-CoA or α-ketoglutarate, which are both intermediates in the TCA cycle. This could indicate that the horses on the F diet had shifted their metabolism towards more aerobic energy which is supported by the lower plasma lactate levels found in response to exercise by Jansson and Lindberg^10^.

The non-essential amino acid asparagine was higher in plasma samples from horses fed the FC diet compared to the F diet. Asparagine is synthesized from aspartate (which also was elevated in plasma in diet FC) and glutamine, where glutamine is converted to glutamate during the reaction^65^. When oxaloacetate from the TCA cycle is converted to aspartate instead of being converted to citrate, asparagine concentrations might rise^65^, therefore, this elevation could be a reflection of the higher protein intake in horses on the FC diet. Asparagine is mainly used for protein synthesis but also contributes to energy production by being back-transformed to oxaloacetate^65,66^.

Carnitine was important for discrimination of the diets, and we found elevated levels in plasma in horses fed diet FC. Carnitine plays a critical role in energy production as it transports long-chain fatty acids into the mitochondria and has previously been found to increase after 2 years of training in Standardbred horses fed a forage-only diet^23^. The uptake of long-chain fatty acids was likely greater in diet FC compared to diet F since total crude fat intake was higher (crude fat content 3–4 times higher in oats compared to grass forage), and an increase of plasma carnitine has previously been observed in humans consuming a high-fat diet^67^.

Another interesting metabolite that was higher in diet F was myo-inositol, a carbohydrate (sugar alcohol) and the most common isomer of inositol in tissue and cells. Inositol is commonly present in plants as phytic acid, and myo-inositol can be synthesized from glucose in the liver and kidneys. Myo-inositol and its phosphorus derivatives are involved in numerous processes, e.g., calcium mobilization, insulin mediator, endocrine modulation, antioxidant processes and are an important growth factor for cell proliferation and survival^68–70^. Oral administration of myo-inositol followed by administration of glucose increased the translocation of GLUT4 transporters on the skeletal muscle cells of mice, which lowered the glucose and insulin concentration in the blood compared to no myo-inositol administration^71^. If the same is true for horses, this could affect energy metabolism as well as increase insulin sensitivity. Myo-inositol has also been found to have an anxiolytic effect in mice and humans, where mice with low anxious-related behaviours had higher plasma concentration of myo-inositol^72^. This is also interesting from a horse perspective since high-starch diets have been linked to anxious behaviour and increased reactivity in horses^14,73^. The lower concentration of myo-inositol could possibly be a factor in this, but this needs further investigation.

The samples analysed in the present study were collected in horses with high energy requirements (double maintenance), low (athletic) body fat content and after the horses had been transported. Horses on diet F had a slightly lower calculated daily energy intake compared to horses on diet FC , however these are theoretical numbers (conversion based on cattle digestion) and no decrease in bodyweight or body condition score was seen during the experiment on diet F. Connysson et al.^9^ investigated, in a cross-over design, the effects of transportation or not prior to exercise on horses fed a forage-only and a forage-oat diet. Transportation elevated cortisol and non-esterified fatty acid concentrations but no changes in acetate, insulin or lactate was found, and the changes followed the same pattern for horses fed the forage-only diet as the forage-oat diet. These alterations reflect a sympathetic process relevant to athletic horses, which are often transported before competitions. Our results are, therefore, of most relevance to athletic horses and further studies on how transportation effects the plasma metabolomic profile would be of interest.

In the present study no washout period was implemented between switching diets, instead diet FC was gradually introduced and a 25 days adaptation period was implemented in both treatments before sample collection. A similar adaptation period is common in feeding trials with horses, e.g. investigating behavioural effects^14^, digestibility, metabolic changes^47,74,75^ and microbial changes^27,28,51^. Although the metabolic separation of diets was evident in our study, one individual did not cluster as tight as the others. Interestingly, this was the same individual that Willing, et al.^2^ observed had consistently (irrespective of diet) and significantly lower diversity of the faecal microbial community. A difference in metabolic response between horses within the same diet is expected since each horse has their individual microbiota, as seen in Willing, et al.^2^, as well as differences in consumption patterns and passage rates. However, by implementing a cross-over study design, we minimize the effects of inter-individual variability and can document the actual dietary effects.

In conclusion, this study complements previous knowledge of changes such as higher plasma acetate concentration in high forage diets and presents new metabolites of interest to further investigate regarding their impact on athletic and competition horse physiology. We found an impact on the host-microbial co-metabolism on diet F with considerably higher plasma concentrations of hippurate and dimethyl sulfone. We suggest that the plasma concentration of hippurate could be further explored as a biomarker for forage intake and health. Furthermore, data support substantial changes in energy metabolism and metabolites like BHB and myo-inositol could be of interest from a performance and behaviour perspective, respectively.

## Supplementary Information

Below is the link to the electronic supplementary material.

Supplementary Material 1

## Data Availability

Data is available upon request from the corresponding author.
